# Analysis of Muscle Load-Sharing in Patients With Lateral Epicondylitis During Endurance Isokinetic Contractions Using Non-linear Prediction

**DOI:** 10.3389/fphys.2019.01185

**Published:** 2019-09-24

**Authors:** Mónica Rojas-Martínez, Joan Francesc Alonso, Mislav Jordanić, Miguel Ángel Mañanas, Joaquim Chaler

**Affiliations:** ^1^Department of Bioengineering, Faculty of Engineering, Universidad El Bosque, Bogotá, Colombia; ^2^Biomedical Research Networking Centre in Bioengineering, Biomaterials and Nanomedicine (CIBER-BBN), Biomedical Engineering Research Centre (CREB), Department of Automatic Control (ESAII), Universitat Politècnica de Catalunya (UPC), Barcelona, Spain; ^3^PM&R Department, Egarsat, Terrassa, Spain; ^4^EUSES-Bellvitge, Universitat de Girona, Universitat de Barcelona, ENTI, Barcelona, Spain

**Keywords:** surface EMG, lateral epicondylitis, non-linear prediction, co-activation, dynamic contraction, endurance

## Abstract

The aim of this paper is to analyze muscle load-sharing in patients with Lateral Epicondylitis during dynamic endurance contractions by means of non-linear prediction of surface EMG signals. The proposed non-linear cross-prediction scheme was used to predict the envelope of an EMG signal and is based on locally linear models built in a lag-embedded Euclidean space. The results were compared with a co-activation index, a common measure based on the activation of a muscle pair. Non-linear prediction revealed changes in muscle coupling, that is load-sharing, over time both in a control group and Lateral Epicondylitis (*p* < 0.05), even when subjects did not report pain at the end of the exercise. These changes were more pronounced in patients, especially in the first part of the exercise and up to 50% of the total endurance time (*p* < 0.05). By contrast, the co-activation index showed no differences between groups. Results reflect the changing nature of muscular activation strategy, presumably because of the mechanisms triggered by fatigue. Strategies differ between controls and patients, pointing to an altered coordination in Lateral Epicondylitis.

## Introduction

Muscular imbalances have been associated with the origin of different musculoskeletal conditions like strain injuries (Yeung et al., 2009), rotator cuff (Lew et al., 2007), low back pain (Nadler et al., 2002), or lateral epicondylitis (Alizadehkhaiyat et al., 2007b; Heales et al., 2016a). They consist in the predominant activation of a muscle or a group of muscles, leading either to overactivation or to disuse that can eventually cause damage to the tissues, especially when concerning repetitive strain injuries (RSI). In this context, previous studies have shown variations in the load-sharing of synergistic muscles associated with pain (Hirata et al., 2015) or fatigue in isometric (Stutzig and Siebert, 2015) and dynamic contractions (Unyó et al., 2013; Smale et al., 2016), altering the initial motor coordination and the adaptation of the control strategy in order to maintain the motor output (Stutzig and Siebert, 2015; Smale et al., 2016).

Lateral Epicondylitis (LE), a common RSI of the forearm, has been associated with repetitive contractions of the extensors in the lateral epicondyle. The pathophysiological nature of this disorder is complex comprising at least three different factors: the local tendon pathology, changes in the pain system and motor system impairments. These lasts are reflected in muscular imbalances (understood as differences in load-sharing and fatigue) and atypical motor control when compared to healthy subjects (Coombes et al., 2009). LE is commonly recognized as being challenging to treat and prone to recurrent episodes (Heales et al., 2016a), hindering the management of the condition (Moore, 2002; Coombes et al., 2009). Thus, improving the identification or monitoring of these muscular imbalances would permit to evaluate patient’s progress during rehabilitation or the design of personalized therapies (Pelletier et al., 2015).

Load sharing between muscles in patients with LE has been studied from the agonist/antagonist strength ratio during dynamic contractions (Unyó et al., 2013). Based on measurements of the exerted torque, Unyó et al. found evidence of muscular imbalances between wrist flexors and extensors in patients with LE, both during isokinetic concentric and eccentric contractions. In addition, when analyzing fatigue during endurance, patients showed a faster decrease in the exerted force compared to controls (Unyó et al., 2013). That is, although all subjects were capable of maintaining the isokinetic exercise at the demanded velocity, the force with which the movement was performed decayed faster in the LE group. Changes at the neuromuscular level, including compensation mechanisms and muscular imbalances, can be better described by the analysis of EMG, which reflect the muscle activation strategy (see for example Page, 2011; Heales et al., 2016a). As such, different studies have identified muscular imbalances in LE during isometric contractions related to grip (Alizadehkhaiyat et al., 2007a; Heales et al., 2016b) and wrist extension (Rojas et al., 2007).

On the other hand, the use of dynamic contractions allows a better insight into the behavior of muscles in normal activities of daily life compared to that obtained from isometric and non-dynamic conditions (Gonzalez-Izal et al., 2010). This kind of analysis is challenging since it is well known that the interpretation of the EMG signal is hampered by the variability induced by the high non-stationary nature of the signal during dynamic conditions, affecting the amplitude and spectral parameters that can be extracted (Rogers and MacIsaac, 2013). In time, alterations in the synergistic activation of muscles during dynamic activities may contribute to pathology (Szucs et al., 2009). Correspondingly, Coombes et al. suggested that management of LE should first identify the factor or the relative expression of factors (tendon pathology, pain and motor impairments) causing the condition so that treatment correspond to its clinical presentation in individual subjects (Coombes et al., 2009).

In this study, we focused on the analysis of the load-sharing between muscles during a dynamic endurance exercise and its relation to LE. Our main hypothesis stated that the co-activation of different muscles would be different for controls and patients during fatigue, as consequence of compensation mechanisms. The identification of alterations in muscles load-sharing may be of use to identify motor impairments, facilitating the management of the condition.

For this purpose, we proposed the application of a non-linear cross-prediction technique previously used by our group for the analysis of respiratory muscles during increased ventilatory effort (Alonso et al., 2011) and that was also validated on a small sample of healthy subjects during dynamic isokinetic exercises (Rojas- Martínez et al., 2013). This technique, which has also been used in recent studies (Tolakanahalli et al., 2015; Vannini et al., 2017), evaluates the coupling between pairs of muscles by predicting the EMG signal of one muscle from the EMG signal of another muscle using lag-embedding and locally linear models.

## Materials and Methods

### Subjects

Twenty subjects participated in the experiment: 10 subjects with no history of musculoskeletal disorders (control group) and 10 patients clinically diagnosed and treated for LE in the past (patient group). Patients were actively using their upper limbs in everyday activities and manual work for at least 6 months, and were free of symptoms or with little discomfort by the time of the experimental session. In this second group, pain was assessed with a Visual Analog Scale (VAS) before and after the experimental protocol.

In order to control for possible variability induced by gender (Nonaka et al., 2006; Brown et al., 2010), all subjects were male, right-handed, and were chosen among those presenting similar age, weight, height, and body mass index in order to have a matched design. No significant differences were found in any of the described indices as analyzed with a Mann- Whitney’s *U* test (see Table 1 for details).

**TABLE 1:** Characteristics of the subjects in the two groups (*N* = 10 each), presented as mean ± standard deviation. Statistical level (*p*) for Mann Whitney’s *U*-test is also presented.

**Characteristic**	**Controls**	**Patients**	***p***
Age (years)	30.3 ± 3.9	33.7 ± 4.6	0.1
Height (cm)	176.1 ± 6.1	178.3 ± 0.1	0.4
Weight (kg)	77.7 ± 7.8	90.1 ± 23	0.3
BMI	25.1 ± 2.3	28.2 ± 6.1	0.2

The study was conducted in accordance with the Declaration of Helsinki and subsequent amendments concerning research in humans and was approved by the Ethics Committee of UPC-BarcelonaTECH and the Spanish Government MINECO in July 19th 2011 with the registration number DPI2011-22680 (“Analysis of the dynamic interactions in non-invasive multichannel biosignals for rehabilitation and therapy”). All volunteers gave their written informed consent to participate.

### Experimental Protocol

**FIGURE 1:**
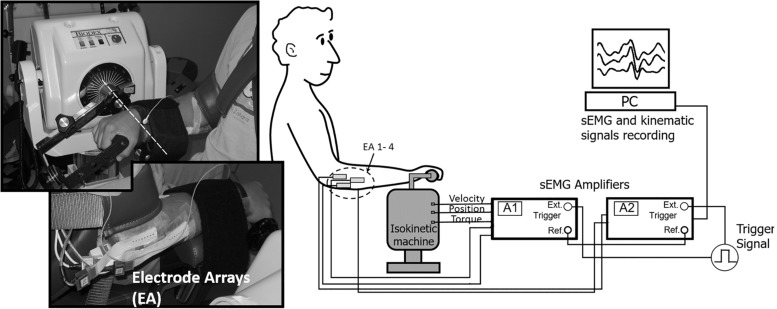
Experimental protocol. **(Left Top)** Position of the subject in the isokinetic machine showing the alignment of the rotational axis with the wrist. **(Left Bottom)** Location of the electrode arrays (EA) for the recording of multichannel EMG signals. Only the location in the extensor muscles can be seen. **(Right)** Schematics of the collection and recording of signals.

Subjects performed a series of concentric wrist extension/flexion exercises on an isokinetic dynamometer (Biodex System III; Biodex Medical Systems, Shirley, NY, United States) up to exhaustion. Subjects were asked to freely perform the exercise at a comfortable level of force, neither too high nor too low, while the instructor gave strong motivation. In all cases, a clear decrease in the exerted toque was observed at the end of the exercise and the subject stopped when he could no longer continue (that is, when fatigue was evident). Subjects were seated with the back straight, elbow flexed at 60°, and the forearm sustained in full pronation. The ulnar styloid was aligned with the rotational axis of the dynamometer and the forearm was fixed with a strap (see Figure 1, left). The velocity of the device was set to 60°/s in wrist extension and 180°/s in wrist flexion in order to emphasize the role of wrist extensor muscles, which are commonly associated with LE (Moore, 2002). The range of motion was 70° (30° in dorsal flexion and 40° in palmar flexion measured from the neutral position of the wrist). The weight of the hand was measured and subtracted at the start of the experimental session for gravity correction of measurements.

Four muscles were analyzed in the study: Extensor Carpi Radialis (ECR), Extensor Digitorum Communis (EDC), Extensor Carpi Ulnaris (ECU) and Flexor Carpi Radialis (FCR). A set of EMG signals were recorded in each of them with a linear array of 8 electrodes. The size of the electrodes in the array was 0.1 by 0.3 mm and were separated by 5 mm (see Figure 1, left). Considering that forearm muscles are difficult to assess with surface EMG, the location of the electrodes was selected according to the procedure described by Mananas et al. (2005): first, the direction of the fibers was drawn over the skin by connecting the origin and insertion of each of the muscles. Then, the subject was asked to exert a selective isometric contraction associated with the muscle under analysis (either, ECR, EDC, ECU or FCR) (Kendall et al., 1993). Finally, the signals were visually inspected for propagation of Motor Unit Action Potentials by displacing a dry linear electrode over the course of the muscle fibers and the best location for each muscle was selected as the one where it was possible to observe the largest and the most similar signals among the different channels of the array. In this location, an adhesive array was fixed for the recording of the signals.

Signals were recorded in single differential configuration, as a voltage between two adjacent electrodes in the electrode array. The reference electrode was located at the wrist and all the signals were amplified, digitized, and sent to the PC for offline analysis by using two sEMG amplifiers with synchronized sampling (16 channel amplifier, model ASE16, LISiN-SEMA Elettronica, Turin, Italy), bandpass filtered (−3 dB bandwidth 10–450 Hz), *F*s = 2048 Hz, 12-bit resolution). The amplifiers support only recording in single differential mode or monopolar mode.

Additionally, the exerted torque, the angular position, and the velocity of movement were measured and sampled at 100 Hz for offline analysis (see Figure 1, right). A trigger signal was used to synchronize the recording of kinematic and EMG signals.

### Data Preprocessing

The obtained EMG signals were filtered between 20 and 350 Hz with a 4th order Butterworth filter following SENIAM recommendations (Freriks and Hermens, 1999). Afterward, a set of double differential (DD) signals were obtained offline by applying a spatial differential filter to reduce crosstalk (Mesin et al., 2009).

**FIGURE 2:**
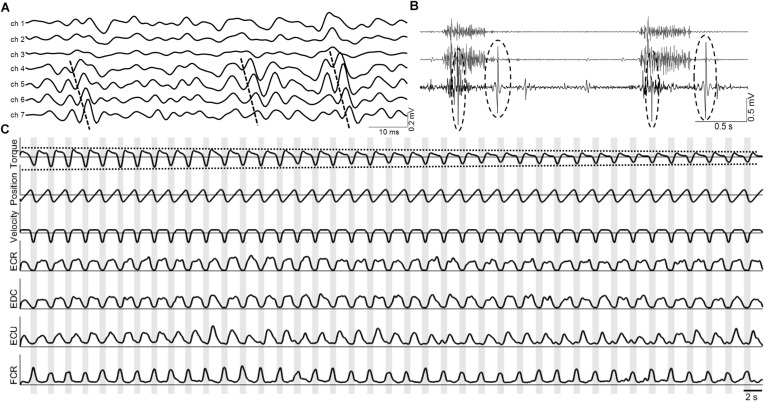
**(A)** Example of the collected multichannel sEMG signals in the ECR muscle. It is possible to see a set of eligible signals for the analysis (ch 4–7), displaying high similarity (i.e., high CC coefficient) and propagating Motor Unit Action Potentials with CV within physiological range (dashed lines). **(B)** Examples of signals presenting movement artifacts (last channel) discarded from the analysis. **(C)** Normalized signal envelopes for torque, position, velocity and ECR, EDC, ECU, and FCR EMG signals. Decrease in the exerted torque is shown in dashed lines. Segments corresponding to flexion are shown in gray shading and were obtained from the zero crossings of the torque signal.

From each pair of adjacent DD signals, the cross-correlation function was calculated. This function was used to calculate the correlation coefficient (CC). The time delay between the adjacent signals in the pair (recorded at a known distance of 5 mm, see section “Experimental Protocol”) was used to estimate the conduction velocity (CV) of the recorded motor unit action potentials. Detailed description of the calculation of CV can be found elsewhere (e.g., Merletti and Parker, 2004). These indices were used to preselect a signal subset (see Figure 2A) among those DD signals presenting a CC > 0.7 and a CV within the physiological range (3 < CV < 7 m/s) (Merletti and Parker, 2004).

Then, the pre-selected signals were visually inspected by an expert for possible movement artifacts and discarded when needed (see Figure 2B). Finally, one DD signal per muscle (the one with the highest CC) was selected and used for the rest of the analysis. In this way, it was possible to guarantee a good signal quality given that EMG signals from the forearm are highly contaminated by crosstalk and are affected by multiple innervation zones, and that the movement velocity (60°/s and 180°/s) can induce large motion artifacts.

Finally, the envelope of each selected EMG signal was obtained by applying full-wave rectification and a 400 ms moving average filter previously applied in cyclic EMG signals (Alonso et al., 2007, 2011). This corresponds to a simple FIR filter with low-pass characteristics without phase distortion. The EMG envelopes were resampled at 20 Hz and normalized to have zero mean and unit variance by subtracting the mean and then dividing by their standard deviation (Alonso et al., 2007, 2011; Rojas- Martínez et al., 2013). In this way, the posterior analysis of their dynamics was independent from their amplitudes (see Figure 2C). The normalization allowed avoiding the effects of the relative location between the electrodes and the origin of the motor unit action potentials on the signal amplitude. In time, this allowed the posterior non-linear coupling to be independent of the amplitude, facilitating its association with the muscular pattern.

On the other hand, torque, angular position, and velocity were used for the analysis of the mechanic and kinematic outputs by estimating the average torque and velocity and the maximum and minimum joint angle across different cycles.

The onset of each cycle (that is, extension followed by flexion of the wrist) was calculated by the zero-crossings of the torque signal.

### Non-linear Cross-Prediction

A non-linear cross-prediction scheme, based on locally linear models built in a lag-embedded Euclidean space (Alonso et al., 2011), was used to assess the coupling between pairs of demodulated EMG signals. This technique uses a delay-based state-space reconstruction and depends on two parameters: the embedding dimension (ED) set to 4, and the delay time (DT) set to a quarter of the duration of a cycle (Alonso et al., 2011; Rojas- Martínez et al., 2013). As the duration of a cycle was approximately 2 s and DT was around 0.5 s, with a sampling frequency of 20 Hz this gave DT values around 10 samples.

The resulting 4-dimensional signal can be expressed as a matrix:

(1) $$S_{t}=\left[\begin{array}{ccc} s_{t} & s_{t+D\,T} & s_{t+2\cdot D\,T}\\ s_{t+1} & s_{t+1+D\,T} & s_{t+1+2\cdot D\,T}\\ \begin{array}{c} \vdots \\ s_{t+P-1} \end{array} & \begin{array}{c} \vdots \\ s_{t+P-1+D\,T} \end{array} & \begin{array}{c} \vdots \\ s_{t+P-1+2\cdot D\,T} \end{array} \end{array}\;\begin{array}{c} s_{t+3\cdot D\,T}\\ s_{t+1+3\cdot D\,T}\\ \begin{array}{c} \vdots \\ s_{t+P-1+3\cdot D\,T} \end{array} \end{array}\right]$$

where *s*_*t*_ represents a sample at time t, *P* is the total number of points in each *ED* calculated as *P* = *NS*−3⋅*DT* and *NS* is the number of samples. Therefore *S*_*t*_ ∈ *ℝ*^*P*×*ED*^, with each row formed by 4 samples spaced *DT* seconds in time.

Couplings between signals were evaluated with the 4-dimensional time series model in Eq. 1 via non-linear cross-prediction based on locally linear models (Sugihara and May, 1990). Theoretically, it is possible to obtain the future value of a point in the embedded space, taking into account only its closest neighbor (and its corresponding future value). In practice, several points have to be considered to build a linear model, that is, an estimation of the tangent hyperplane which approximates the global non-linear dynamics at the point of interest. In this work, 3 ⋅ *ED* = 12 nearest neighbors were used to obtain reliable prediction values (Alonso et al., 2007, 2011).

Then, the models mapping the embedded signal into the future (after a prediction horizon, PH) to predict a different embedded signal (cross-prediction scheme) were obtained using a leaving-one-out approach. Each model was obtained *P* times for each value of *PH* for each EMG-EMG pair to study changes in non-linear muscle coupling. The goodness of the prediction was evaluated by the *R*^2^ coefficient as a function of *PH:* the higher the coefficient the higher the coupling between muscles. For more details on the actual prediction algorithm, see the Annex, or please refer to Sugihara and May (1990) or Alonso et al. (2011).

Three different analyses were performed: The first considered the analysis of the prediction taking into account the entire length of the exercise, that is, *NS* corresponded to the total number of signal samples.

In the second analysis the test was divided into three segments at the beginning, middle, and final stages of the exercise, each of them comprising 15 cycles, that is, *NS = 15⋅2 s⋅20 samples/s = 600 samples* (Rojas- Martínez et al., 2013). Given that subjects were asked to complete the task up to exhaustion, the mentioned segments were overlapped in those cases where the subject completed less than 45 cycles. In these two analyses the area under *R*^2^ up to a *PH* = 10 s was used as an index for the characterization of the *PH* vs. *R*^2^ curves. Then, the cross-prediction between initial and middle, middle and final, and initial and final segments was compared for each group separately (controls or patients). In this context, such an index could be considered as a measure of the *average* performance of the cross-prediction up to five cycles (*PH* = 10 s).

The third analysis, unlike the previous two, was intended to evaluate changes in predictability on a cycle-by-cycle basis. For this purpose, the first 15 cycles of a signal recorded in a given muscle were used to predict a signal segment recorded in another muscle. This last segment corresponded to a sliding window of 15 cycles of duration that advanced one cycle at a time from the beginning to the end of the contraction. In other words, from the signal of a given muscle at the beginning of the contraction, it was possible to progressively assess the prediction of the activation of another muscle one or more cycles later. The initial segment of the first signal was always used to predict a sliding segment of a second one, making possible to assess the predictability course as the exercise progresses. In this case, the short-term prediction was characterized by the area under *R*^2^ up to 0.5 s (10 samples) as the prediction horizon was implicitly considered in the moving window of the second signal. In order to avoid possible bias in the course of predictability due to different initial values of different cross-prediction pairs, this measure was normalized with respect to its initial value.

### Co-activation Index

Couplings between muscles, estimated using non-linear cross-prediction, were compared with a commonly used muscle co-activation index. This index represents the common work performed by a pair of muscles with respect to their total activation during the execution of a given task. In this paper, the co-activation index between muscles *a* and *b* was calculated as (Winter, 2009; Davidson et al., 2013).

(2) $$C\,I_{a\,b}=2\times \frac{\int_{t_{o}=0}^{t_{f}=100\%\,D\,C}\min\left(n\,E\,M\,G_{a},n\,E\,M\,G_{b}\right)\,d\,t}{i\,E\,M\,G_{a}+i\,E\,M\,G_{b}}\times 100$$

where *a* and *b* can be any of the signals belonging to the set of muscles *{ECR, EDC, ECU, FCR}*, *DC* is the duration of each cycle as determined by the zero crossings of the torque signal. The term *iEMG* corresponded to the integrated value of the signal calculated as:

(3) $$i\,E\,M\,G=\int_{t_{o}=0}^{t_{f=100\%\,D\,C}}n\,E\,M\,G\,d\,t$$

where *nEMG* is the envelope of the EMG signal normalized with respect to its standard deviation for each cycle as in Davidson et al. (2013). This normalization allows avoiding interindividual variability and bias due to differences in signal amplitudes caused by factors such as the distance between the recording electrode and the source.

### Statistical Analysis

Data was tested for normality with the Kolmogorov-Smirnov test and it was found that in most cases it did not follow a normal distribution. Besides, considering that the size of the sample was small (*N* = 20), non-parametric tests were used to assess intra or inter-group differences. Specifically, the Wilcoxon signed rank test in the former case and the Mann-Whitney’s *U* test in the latter. Differences were considered significant at *p* = 0.05.

## Results

Subjects in the group of patients scored a VAS of 1.73 ± 1.67 and 2.52 ± 2.21 before and after the experimental protocol, respectively. No significant differences between the initial and final VAS score were found (*p* = 0.07 with a Wilcoxon’s signed-rank test).

### Kinematics and Torque

Table 2 shows the velocity during extension and flexion, the range of movement, the duration of each cycle, and the torque ratio between initial and final cycle for the two groups in mean and standard deviation. No significant differences in the average value of any of these variables were observed (*p* > 0.1, either between subjects or between groups). Additionally, their variability between different cycles showed no differences between the two groups (*p* > 0.4 in all cases except for velocity during flexion in both cases).

**TABLE 2:** Kinematic and mechanical output for the two groups.

	**Control group**	**Patient group**	***p* (between subjects)**	***p* (within subject)**
Velocity_extension_ (º/s)	59.1 ± 2.1	58.7 ± 2.7	0.4	0.4
Velocity_flexion_ (º/s)	181.8 ± 6.2	179.8 ± 7.3	0.003	0.02
Range (º)	70.0 ± 1.0	69.3 ± 0.7	0.3	0.8
Duration (s)	2.1 ± 0.2	2.2 ± 0.2	0.1	0.4
Torque_final/initial_	0.3 ± 0.1	0.4 ± 0.2	0.4	–

Regarding the exerted torque, it is possible to see from variable *Torque_final/initial_* that it decreased at least 50% of its initial value at the end of the exercise, either for controls or patients (*p* < 0.002 for a Wilcoxon test).

### Prediction Considering the Entire Duration of the Exercise

The R^2^ coefficient as a function of PH was calculated for all prediction models (ECR-EDC, ECR-ECU, ECR-FCR, EDC-ECU, EDC-FCR, and ECU-FCR). The area under *R*^2^ for controls and patients (mean ± standard deviation) is shown in Table 3. The statistical significance obtained through a Mann-Whitney’s *U* test is also presented. It is possible to observe that significant differences between the two groups were obtained only for two of the three models that considered the prediction from the signal corresponding to *extensor carpi radialis* (ECR-ECU and ECR-FCR). Hence, in the rest of analyses only the couplings between ECR and other muscles (that is, ECR-ECU, ECR-FCR, ECR-EDC) will be taken into account.

**TABLE 3:** Area under the curve for the different cross-prediction models.

	**Controls**	**Patients**	***p***
ECR-EDC	79.8 ± 7.7	74.4 ± 6.7	0.2
ECR-ECU	74.5 ± 8.1	65.3 ± 7.6	0.02^∗^
ECR-FCR	80.3 ± 8.4	71.2 ± 8.6	0.04^∗^
EDC-ECU	67.8 ± 14.3	62.7 ± 12.7	0.4
EDC-FCR	76.6 ± 7.1	70.7 ± 11.5	0.2
ECU-FCR	73.9 ± 10.3	70.3 ± 12.2	0.5

*Results are presented for both groups as mean and standard deviation. The significance value (p) for the comparison between the groups is also shown. ^∗^Denotes statistical significance obtained using Mann- Whitney’s *U* test.*

**FIGURE 3:**
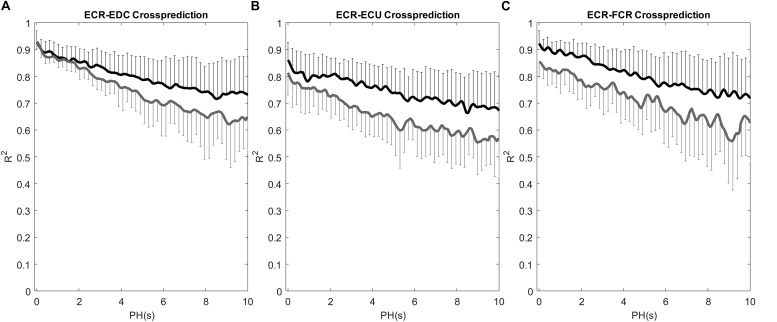
*R*^2^ coefficient as function of PH for the cross- prediction models: **(A)** ECR-EDC, **(B)** ECR-ECU and **(C)** ECR-FCR. Each point represents the mean and standard deviation for controls (black) and patients (gray).

Figure 3 shows the course of R^2^ as a function of PH for these pair of muscles. Results are presented as mean and standard deviation for both groups with a resolution of 50 ms. It is possible to observe a different behavior between groups displaying a better prediction for controls (black traces) than for patients (gray traces) or, in other words, controls presented a higher predictability over the whole range of considered PH.

### Prediction at Different Stages of the Exercise

**FIGURE 4:**
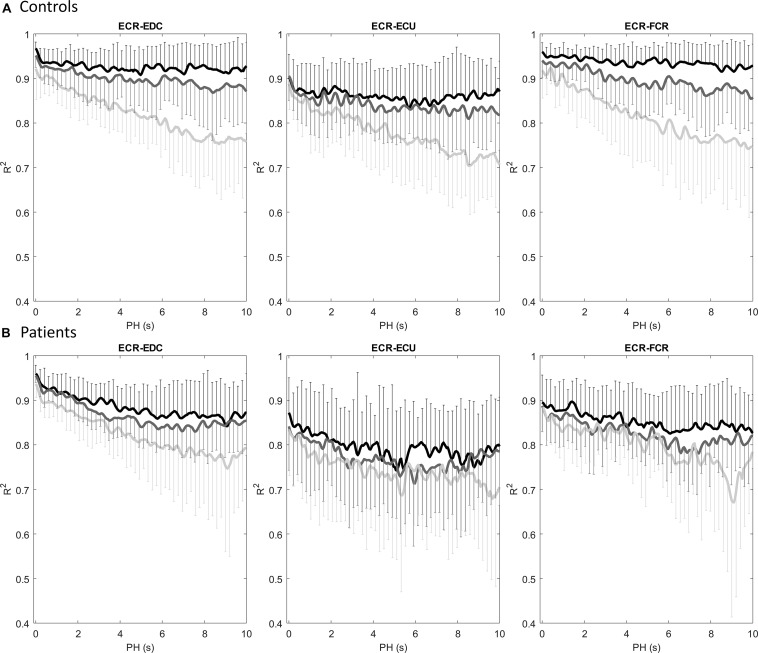
*R*^2^ as function of PH for different segments of the exercise: initial (black), middle (dark gray) and final (light gray). Results are presented as mean and standard deviation for: **(A)** controls, and **(B)** patients.

*R*^2^ as a function of *PH* for the three segments at the initial, middle and final stages of the exercise and for the different models is shown in Figure 4. Results are presented for each group separately. It is possible to observe that in the case of controls, the *R*^2^ coefficient displayed higher values than in patients at the initial segment (except for very low values of PH in the ECR-EDC pair), displaying a low decrease with increasing *PH*. In the middle and, especially at the end of the exercise, the *R*^2^ coefficient decreased faster, showing a weaker coupling between muscles as the exercise advanced. However, *R*^2^ in patients showed a fast decrease right from the beginning, displaying similar trends for each of the segments (initial, middle or final) independently of the model (ECR-EDC, ECR-ECU or ECR- FCR). Therefore, the cross- prediction between muscles is weaker in patients than in controls regardless of the model, probably because of a lack of coordination between muscle pairs. Differences between segments for each group were assessed quantitatively from the area under *R*^2^ with a paired-sample Wilcoxon test. Results are displayed in Table 4 for each group separately: for controls, the area under *R*^2^ was higher at the initial and middle stages than at the final stage of the exercise (*p* < 0.05 for all cross-prediction models). Additionally, the area under *R*^2^ at the middle stage was lower than at the beginning of the exercise for all cross-prediction models (*p* < 0.01), except for ECR-ECU. No statistical differences were found in the group of patients. On the other hand, coupling between ECR (understood as the area under *R*^2^) and the other three muscles was significantly lower in patients than in controls at the beginning of the exercise (see Table 5). Such differences remained at the middle stage but only for the pairs ECR-ECU and ECR-FCR, which interestingly were the muscle pairs that showed significant differences between the groups when the whole exercise was taken into account (see Table 3 in the previous section).

**TABLE 4:** Statistical significance for the comparison of the area under *R*^2^ between different segments.

	**Initial-Middle**		**Middle-Final**		**Initial-Final**	
			
	**Controls**	**Patients**	**Controls**	**Patients**	**Controls**	**Patients**
ECR-EDC	0.01^∗^	0.4	0.004^∗^	0.2	0.002^∗^	0.1
ECR-ECU	0.13	0.5	0.049^∗^	0.5	0.01^∗^	0.4
ECR-FCR	0.002^∗^	0.4	0.01^∗^	0.6	0.002^∗^	0.1

*Results are presented for each group separately. ^∗^Denotes statistical significance for Wilcoxon test.*

**TABLE 5:** Statistical significance for the comparison of the area under *R*^2^ between the two groups for the different segments of the test and models.

	**ECR-EDC**	**ECR-ECU**	**ECR-FCR**
Initial	0.04^∗^	0.05^∗^	0.01^∗^
Middle	0.12	0.04^∗^	0.01^∗^
Final	0.91	0.74	0.85

*^∗^ Denotes statistical significance for Mann- Whitney’s *U* test.*

### Cycle by Cycle Cross-Prediction

**FIGURE 5:**
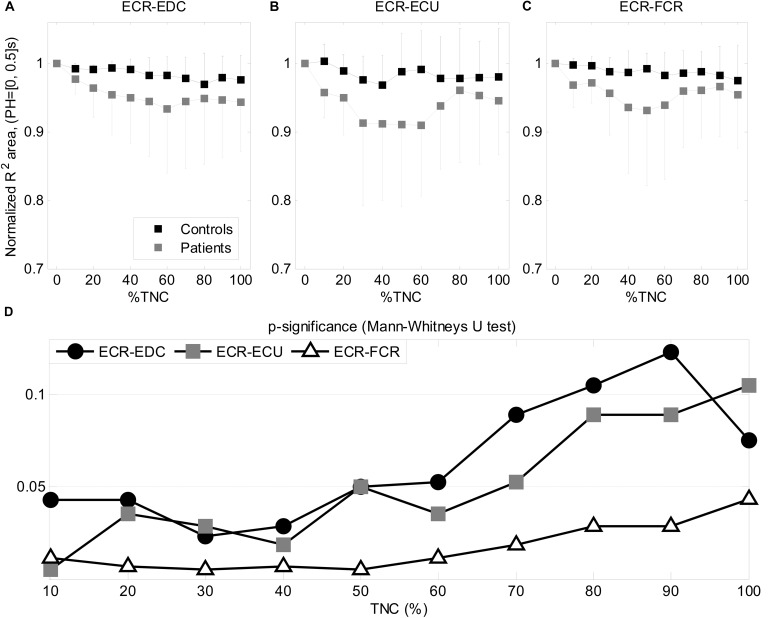
Cross-prediction during exercise. The area under *R*^2^ for three models **(A–C)** are displayed as function of the total number of cycles (TNC). The statistical significance obtained after the comparison between groups is shown as function of TNC **(D)**.

Figures 5A–C show the index describing the area under *R*^2^ (up to *PH* = 0.5 s) as obtained from the cross-prediction models using a sliding window across the Total Number of Cycles (%TNC) for each subject. This index was normalized with respect to its initial value at 0%TNC in order to focus on changes in muscle cross-prediction (i.e., coupling) over time. It is possible to observe a higher drop in patients (gray) than in controls (black), meaning that the cross-prediction decayed faster in patients. Such differences can be observed from the area under the *R*^2^ curve. Figure 5D shows the significance obtained from the quantitative comparison of the cumulative area between the two groups every 10% TNC for each of the models (ECR- EDC, ECR-ECU, ECR- FCR) using a Mann-Whitney’s *U* test. ECR-FCR coupling decayed faster in patients for all TNC (*p* < 0.05). Although the coupling between ECR and the muscles EDC and ECU decayed more rapidly than coupling between ECR and FCR, it was noticeable only up to the first half of the test (50% TNC), showing no significant differences afterward.

### Co-activation Index

The co-activation index (CI) in Eq. 2 was calculated in the same way as in the cross-prediction analyses in order to compare the coupling between muscles as obtained with one or the other index. The *CI* between ECR and the other muscles for the entire duration of the exercise and for both groups (mean ± standard deviation) is presented in Table 6. Additionally, Table 7 shows *CI* at the initial, middle, and final segments considering 15 extension/flexion cycles for each stage during the exercise. Unlike for the cross-prediction analysis, no significant differences between the groups were found in any case for *CI*, that is, neither for the total duration of the exercise nor when considering segments.

**TABLE 6:** Co-activation index (%) for the entire duration of the test.

	**Controls**	**Patients**	***p***
ECR-EDC	86.7 ± 2.95	87.6 ± 3.86	0.5
ECR-ECU	72 ± 9.94	73.7 ± 10.1	0.8
ECR-FCR	43.6 ± 7.56	50.8 ± 7.69	0.08

*Statistical significance between groups was calculated using the Mann-Whitney’s *U* test.*

**TABLE 7:** Co-activation index (%) for three test segments at the initial, middle and final stages of the test and statistical significance between segments (*p*) for the Mann-Whitney’s *U* test.

	**Initial**			**Middle**			**Final**		
			
	**Controls**	**Patients**	***p***	**Controls**	**Patients**	***p***	**Controls**	**Patients**	***p***
ECR-EDC	88.1 ± 3.8	88 ± 4.3	1	87 ± 3.9	87.1 ± 4.8	1	86 ± 3.9	86.8 ± 4.8	0.7
ECR-ECU	69.7 ± 15.2	71.9 ± 9.1	1	69.4 ± 12.6	72.8 ± 10.8	0.6	69.8 ± 12.6	72.1 ± 12.1	0.6
ECR-FCR	40.7 ± 10.2	50.1 ± 8.3	0.06	44.1 ± 10.1	51.7 ± 8.8	0.06	43.9 ± 8.6	51.6 ± 8.8	0.06

**FIGURE 6:**
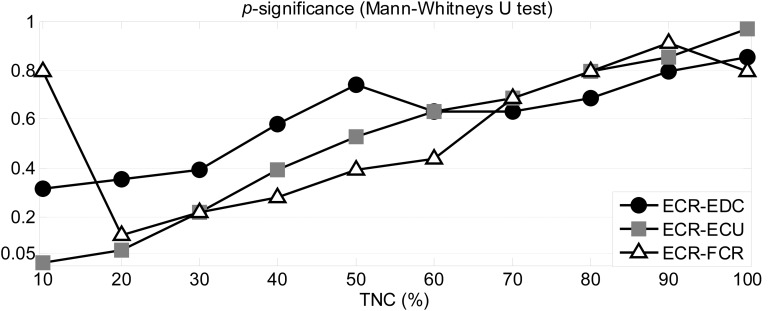
Statistical significance for the comparison between patients and controls when analyzing changes in the CI along the exercise for the different pairs of muscles.

The statistical significance obtained for the comparison between the three segments is shown in Table 8 for each group separately where it was possible to observe differences between segments for all muscle pairs in controls (see Table 4). *CI* showed significant differences only for: 1) the ECR-EDC pair when comparing the initial and middle segments with the final one, and 2) the pair ECR-FCR when comparing the initial with the final segment. Finally, changes in the *CI* were also assessed cycle by cycle using a moving window of 15 cycles. Figure 6 displays the significance for Mann-Whitney’s U test when comparing the cumulative *CI* between groups every 10%TNC (from 10 to 100%TNC). *CI* was normalized with respect to its initial value following the same methodology as in the cross-prediction analysis. Contrary to the previous analysis (see Figure 5), no significant differences using *CI* were obtained but for the pair ECR-ECU at 10% TNC.

**TABLE 8:** Statistical significance for the comparison of CI between segments of the exercise for controls and patients.

	**Initial-Middle**		**Middle-Final**		**Initial-Final**	
			
	**Controls**	**Patients**	**Controls**	**Patients**	**Controls**	**Patients**
ECR-EDC	0.2	0.2	0.001^∗^	0.3	0.04^∗^	0.3
ECR-ECU	0.7	1	1	0.4	0.9	0.6
ECR-FCR	0.06	0.2	0.06	0.9	0.04^∗^	0.2

*^∗^Denotes statistical significance for Wilcoxon’s test.*

## Discussion

### Analysis of the Total Duration of the Test

Results obtained for the entire duration of the test revealed a lower predictability for patients than for healthy subjects only in those pairs involving the ECR (*p* < 0.04, Table 3). Muscle coupling between the ECR decreased as the prediction horizon PH increased, and this trend was more pronounced in patients than in controls even when no evident changes were observed in the signals (see Figure 2C as an example and Table 3 for differences between groups). In contrast, the co-activation index, considered as the gold standard for the evaluation of changes in load- sharing [see for example (Bekkers et al., 2018; Richards et al., 2019)], did not show any significant difference between groups (see Table 6).

In this respect, it is important to note that although the two methods yielded different results, its comparison can be limited by the fact that the co-activation index may not be capable of following the dynamics of the EMG signal. Therefore, it is important to compare the results obtained by non-linear prediction with other signal processing techniques lacking of this limitation, such as muscle synergy assessment based on matrix factorization [see for example (Tresch et al., 2006; Ebied et al., 2018)].

### Analysis at Three Stages of the Test

When the exercise was divided into initial, middle and final stages, the Mann-Whitney’s *U* test revealed several differences between the two groups at the initial and medium stages for all of the selected pairs of muscles: ECR-EDC, ECR-ECU, and ECR-FCR (Table 5). In addition, in comparison to the control group, patients evidenced a reduced predictability since the beginning of the contraction, showing a similar decay across different segments.

With respect to changes between the three stages, significant differences between segments were found for the control group (see Table 4), both between initial and middle segments (*p* < 0.05 for ECR-EDC and ECR-FCR) or initial and final segments (*p* < 0.05 in all cases). This can be associated to a higher variability in non-linear coupling throughout the duration of the endurance test in this group. Additionally, long-term muscular predictability (up to 10 s, approximately 5 duty cycles) is higher for the group of controls at the beginning of the exercise for all muscle pairs (Table 5). As the exercise progresses, patients and controls show a similar behavior, displaying no differences at the final segment (see Figure 4 and Table 5). On the other hand, patients showed reduced predictability from the beginning of the test, with a similar trend for different stages of the exercise showing no significant differences between segments (Table 4). Hence, the predictability pattern is low in patients throughout the exercise displaying a lower coupling between the ECR and the other muscles. This can be associated with an earlier manifestation of fatigue in this muscle.

Like the cross- prediction assessment, the co-activation index in controls revealed significant differences between stages of the exercise but not in all cases: only between the initial and final or middle and final segments for the ECR-EDC pair and between the initial and final segments for the ECR-FCR pair (see Table 8). What is more important, contrary to the cross-prediction analysis, the co-activation index failed to find differences in muscle coupling between controls and patients, independently of the stage of the test (see Tables 4, 7).

### Changes During the Test

The analysis with a sliding window of 15 cycles allowed the evaluation of short-term predictability changes (Figure 5). Unlike the previous analyses, such evaluation did not depend on the actual values of the studied measures (area under *R*^2^ or co-activation index) but was rather focused on their change rate over the total number of cycles (TNC). The non-linear cross-prediction analysis showed differences between groups up to 50% TNC for ECR-EDC and ECR-ECU and for the total length of the test for ECR-FCR, indicating higher drops in prediction for patients than for healthy subjects. Therefore, in addition to what can be interpreted as a poorer muscle balance in patients according to results in previous sections, such imbalances were more evident for short-term predictability. This behavior was not observed from the rate of change of the co-activation index (see Figure 6). Thus, the change-rate of the proposed predictability measure on a cycle by cycle basis evidenced different muscular patterns elicited by controls and patients, especially at the beginning of the exercise and until approximately half of its total duration.

In general, the load-sharing between four forearm muscles, namely *Extensor Carpi Radialis* (ECR), *Extensor Digitorum Communis* (EDC), *Extensor Carpi Ulnaris* (ECU), and *Extensor Carpi Radialis* (ECR), was assessed during a dynamic endurance exercise in controls and patients with lateral epicondylitis (LE). The non-linear cross-prediction analysis allowed the assessment of coupling between muscles at different time-scales: the higher the *R*^2^ value, the higher the coupling between the considered EMG signals. Given that the predictability is a measure of correlation between data, this analysis can also be interpreted as a way of quantifying the degree of coordination between muscles, or, more generally, a measure of dependence between muscular activity.

Analysis of kinematic data showed that healthy subjects were able to maintain the target velocity and to perform the movement of the wrist over the whole predefined range of motion up to the end of the exercise, even after the decline of the muscular mechanical output caused by fatigue (decrease of the initial torque, see Table 2).

Although the kinematic output was not altered by fatigue, the performed analysis showed differences between controls and patients with LE. Results indicate that the coordination between muscles during the endurance conditions was changing and, therefore, their load-sharing. The predictability decreased over time in both groups, reflecting the changing nature of muscular control strategy.

The presented findings showed that activation strategies also differ between controls and LE patients, pointing to muscle imbalances affecting the ECR in the latter. Given that the EMG signals were normalized, the findings cannot be attributed to methodological differences in the amplitude of the signals, nor can be known if the contribution of the ECR to the contraction increased or decreased with time. However, the presented findings are consistent with different studies. For example, Heales et al. reported an altered activation of ECR, EDC and Flexor Digitorum Profundus in LE patients during a low effort isometric contraction. In that case, the ECR exhibited a lesser activation during the contraction in the symptomatic arm. Interestingly, altered activations were also found in the asymptomatic arm exhibiting bilateral control changes (Heales et al., 2016b). Similarly, a different study reported differences in the co-activation of muscles during an isometric endurance task, displaying a reduced activity of the ECR at the end of the contraction (Alizadehkhaiyat et al., 2007b). In addition, Mista et al. (2018) in concluded that there is a reorganization of force control strategies in chronic LE patients based on the analysis of isometric contractions. On the other hand, previous studies had evidenced a higher fatigability of the ECR in LE during isometric contractions (Rojas et al., 2007). Consequently, it is reasonable to suggest that the observed alterations in muscle coupling affecting particularly the ECR, can be attributed to fatigue although further studies are needed.

Szucs et al. also observed changes in muscle load sharing as consequence of a fatiguing dynamic exercise affecting the activation of the upper trapezius (Szucs et al., 2009). As pointed out by the authors, the identification of alterations in muscle activation during dynamic contractions may be valuable to decide clinical interventions directed to shoulder pathologies. The same rationale could be applied to Lateral Epicondylitis as suggested by Coombes et al. (2009) that proposed that therapies intended for LE should take into account alterations in motor control. This last is also supported by findings of cortical changes in different musculoskeletal disorders, including LE, that need to be properly addressed to guarantee recovery (Pelletier et al., 2015).

Lateral Epicondylitis has been assessed in literature mostly during isometric static contractions that represent poorly the activation of muscles in daily life activities. With this respect, different authors have supported the use of dynamic- isokinetic contractions, which are more representative, in order to monitor and manage different musculo-skeletal disorders (Croisier et al., 2007; Sahin et al., 2011; Alizadehkhaiyat and Frostick, 2015). In this context, the methodology proposed in the present study can provide a way to analyze muscle coupling (load sharing) during dynamic contractions, providing deeper insight into muscle activation compared to isometric contractions.

In summary, results in the present study are consistent with previous studies showing that the activation of ECR is altered in LE. What is more, findings show that coupling between ECR and other forearm muscles rapidly decrease in LE both at short and long-terms during dynamic tasks.

The observation of an altered activation of the ECR could be of great value for designing personalized (namely specific strengthening programs) rehabilitation protocols and assess their outcomes, favoring secondary prevention. In fact, secondary prevention is a major concern in epicondylitis since symptom relapse is very high (8.5% in the first 2 years) (Sanders et al., 2015), especially in work related injury patients. Compensation mechanisms regarding the role of ECR should be taken into account. Particularly, rehabilitation exercise strategies should focus on maintaining a balanced activation pattern of the ECR, taking into account not only its performance but also its interaction with other extensors and flexors of the wrist. This kind of monitoring can be done by providing biofeedback based on the continuous evaluation of muscle coupling from surface EMG. In addition, the proposed methodology can potentially serve to detect effectively pathophysiological changes related to epicondylitis and to assess the effectiveness of treatments based on quantitative indexes. In addition to the probable contributions to the design of intervention therapies, the altered activation may be an indicator of risk factor providing highly relevant information to ergonomists in order to organize preventive plans in susceptible subjects. However, further longitudinal studies should be designed to prove that.

Limitations of the study include the effect of crosstalk in the recorded EMG and the continuous evaluation of pain. Crosstalk presents a serious issue in the recording of EMG in the forearm due to the activation of narrow and closely spaced muscles. In order to address this issue, Double Differential signals were computed and used in the analysis (Mesin et al., 2009). In addition, the linear arrays used in this study comprised small electrodes (0.1 × 0.3 mm) and small inter-electrode distance (0.5 mm) as recommended to avoid crosstalk (Yung and Wells, 2013). Other techniques to suppress crosstalk, such as high-density electromyography can be considered in future studies (Talib et al., 2019).

Given that patients did not report significant increase in pain after the experimental protocol, the observed changes may be attributed to fatigue. Previous findings on alterations of motor control in the asymptomatic limb of patients with unilateral presentation of LE also support this statement (Heales et al., 2016b). However, future studies should consider the evaluation of pain during the task to confirm that the presented findings are not correlated to this factor.

In addition, it has also been observed that musculoskeletal disorders like Lateral Epicondylitis induce changes in the cortical representation of the muscles (Pelletier et al., 2015; Schabrun et al., 2015). It has been suggested that those changes may persist even after symptoms (pain) disappear (Pelletier et al., 2015). Aforementioned reorganization of forearm muscles in the cortex can explain the alterations in load-sharing during fatigue in LE, observed in this study.

## Conclusion

A non-linear cross-prediction scheme based on locally linear models was used to quantify the difference in muscle co-activation between controls and patients with lateral epicondylitis during endurance contractions. Results showed a particularly notable difference in co-activation between Extensor Carpi Radialis and the other analyzed forearm muscles. This finding suggests that control strategy of this muscle is particularly affected by the condition, which is consistent with the findings of other studies available in the literature. It was also demonstrated that the proposed method is more sensitive to the slight differences in the activation of synergistic muscles than the co-activation index, a method commonly used in the literature.

The proposed methodology could find its application in clinical practice, i.e., in assessment of the state of the patient and the design and evaluation of the effectiveness of treatments based on the derived quantitative indexes from surface electromyography rather than in subjective feedback, such as the evaluation of pain or functional limitations of the upper limb.

In future works, (i) more patients should be included in the study and a longitudinal design must be adopted to confirm the results, (ii) the relation between pain during the execution of the tasks and its incidence in the load sharing of muscles should be analyzed, (iii) recordings could be repeated using high-density electromyography, as a more advanced recording technology and, (iv) more sophisticated statistical techniques to analyze the dynamic of the EMG signal will be applied.

## Annex: Cross-Prediction and Delay-Based State-Space Reconstruction

In general, the dynamics of a system can be explained by several underlying variables, which are not available from outside or are not easily or directly measurable. These variables affect the state of a system, which in turn reflects on some other quantities that can be more easily recorded, such as surface EMG signals.

**FIGURE 7:**
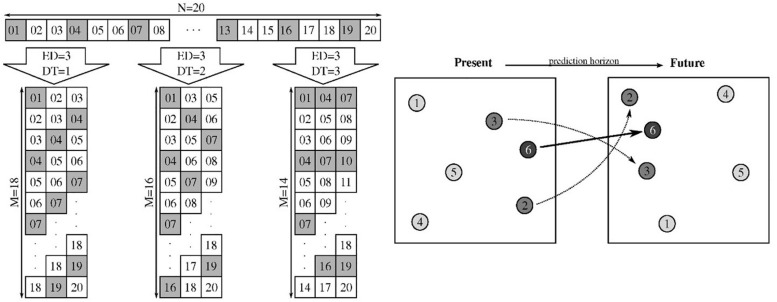
**(Left)** Examples of delay embedding for a time series of length *N* = 20. Three different embeddings with ED = 3 are depicted: *DT* = 1, 2, and 3. Some samples of the time series are gray-shaded to act as reference points when looking at the embedded matrix. Reprinted from Alonso et al. (2011), with permission from Elsevier. **(Right)** Graphical example of local linear prediction using *ED* = 2 and 2 neighbors. Five points (1–5) with known images in the future are shown, whereas image of point 6 after a certain prediction horizon is unknown. Using images of the 2 nearest neighbors (points 2 and 3), forecast for point 6 is obtained. Reprinted from Alonso et al. (2011), with permission from Elsevier.

Delay-based state-space reconstruction, also known as delay-embedding, allows a representation of the state of the system based on sequences of time-lagged data points (Sugihara and May, 1990). These sequences depend only on the embedding dimension (ED) and the delay time (DT), and their values depend on the application. See several examples in Figure 7 (left).

By embedding a scalar time series we obtain vector time series of multidimensional points that represent the state of the system along time (Takens, 1981; Kaplan and Glass, 1995). This ED-dimensional time series can be used to predict the future of a point after some time (prediction horizon). Theoretically, the future image of the present nearest neighbor could be taken as a predicted value of the point of interest, but uncertainties in the signal recording produced by noise and quantization errors require all nearest neighbors inside a small-enough hypersphere to be considered. With these neighbors, a locally linear equation system can be solved easily by least-squares to predict our point after the prediction horizon (see a two-dimensional example in Figure 7, right). The use of multiple locally linear models stitched together produces a global non-linear regression model that constitutes an estimation of the underlying non-linear dynamics of the system.

For the analysis of EMG envelopes in the current work, the embedding dimension was set to 4 and the delay time to a quarter of exercising cycle, in a way that each 4-dimensional point contains information of the whole cycle. Moreover, non-linear prediction was performed in a leaving-one-out fashion, that is, each global regression model was calculated as many times as points there were, each time leaving a single data point out.

## Data Availability Statement

The raw datasets generated for this study can be available on request to the corresponding author.

## Ethics Statement

The study was conducted in accordance with the Declaration of Helsinki and subsequent amendments concerning research in humans and was approved by the Ethics Committee of UPC-BarcelonaTECH and the Spanish Government MINECO in July 19th 2011 with the registration number DPI2011-22680 (Analysis of the dynamic interactions in non-invasive multichannel biosignals for rehabilitation and therapy). All volunteers gave their written informed consent to participate.

## Author Contributions

MR-M, JC, and MM conceived and designed the experimental protocol and conducted the experiments. MR-M, JA, and MM designed the study and interpreted the results. MR-M was in charge of the implementation of signal processing and the analysis of the data with the aid of JA. MJ aided in the analysis of the data and in the interpretation of results. JC aided in the interpretation of results. MR-M and JA wrote the manuscript. All authors contributed to revising the manuscript.

## Conflict of Interest

The authors declare that the research was conducted in the absence of any commercial or financial relationships that could be construed as a potential conflict of interest.
